# Identification of IL1RN, MUC1, and SERPINA1 as key NET-related biomarkers in ulcerative colitis-associated intestinal fibrosis via bioinformatics and experimental validation

**DOI:** 10.1186/s12876-026-05179-5

**Published:** 2026-07-31

**Authors:** Qiang Chuai, Xiaohong Jiang, Kaixuan Li, Xin Liu, Jia Guo, Jianping Liu

**Affiliations:** 1 Graduate School, Hebei University of Chinese Medicine, Shijiazhuang, Hebei China; 2 Dongfang Hospital, Beijing University of Chinese Medicine (Qinhuangdao Traditional Chinese Medicine Hospital), Qinhuangdao, Hebei China; 3 Department of Gastroenterology, The First Affiliated Hospital of Hebei University of Chinese Medicine, Shijiazhuang, Hebei China

**Keywords:** Ulcerative colitis-associated intestinal fibrosis, Neutrophil extracellular traps, Bioinformatics, Machine learning

## Abstract

**Background:**

Long-term recurrent episodes of ulcerative colitis (UC) can lead to intestinal fibrosis (IF), which severely affects patient prognosis. Neutrophil extracellular traps (NETs) have been identified as critical drivers of various fibrotic diseases; however, their precise role and regulatory mechanisms in UC-associated intestinal fibrosis (UC-IF) remain to be systematically elucidated. Therefore, identifying the key regulatory genes through which NETs participate in UC-IF is highly important for the diagnosis and treatment of this condition.

**Methods:**

In this study, we analyzed the gene expression profiles of UC patients via publicly available databases and screened for neutrophil extracellular trap-related differentially expressed genes (NET-DEGs), identifying a total of 99 NET-DEGs. Enrichment analysis was then performed to explore the biological processes involving these genes. Furthermore, this study identified 5 potentially key NET-DEGs using machine learning algorithms. On the basis of these 5 genes, combined with protein-protein interaction (PPI) network analysis, intestinal fibrosis-related gene set variation analysis (GSVA), and immune infiltration analysis, we identified NET-related biomarkers associated with UC-IF. Finally, we established a mouse model of UC-IF in vivo to validate the bioinformatics findings.

**Results:**

This analysis revealed that IL1RN, MUC1, and SERPINA1 represent a novel panel of NET-related biomarkers for UC-IF. These genes were enriched in immune responses, interleukin signaling pathways, O-glycan biosynthesis, and mucosal barrier-related pathways. Furthermore, these genes were positively correlated with several immune cell types, including neutrophils, macrophages (M0 and M1), and dendritic cells, collectively mediating immune dysregulation in UC and thereby contributing to the pathogenesis of UC-IF. Notably, animal experiments further validated the aberrantly high expression of these genes in the intestinal tissues of UC-IF mice, which was consistent with the bioinformatics findings.

**Conclusions:**

As UC-IF-associated NET biomarkers, IL1RN, MUC1 and SERPINA1 provide novel insights into the clinical diagnosis, treatment, and translational research of UC-IF.

**Supplementary Information:**

The online version contains supplementary material available at 10.1186/s12876-026-05179-5.

## Introduction

Ulcerative colitis (UC) is a lifelong intestinal disorder [1]. Its global prevalence has been steadily rising [2]. In China, the prevalence of UC has also increased sharply, transforming UC from a rare gastrointestinal disease into a commonly encountered one. Given the country’s vast population, the total number of UC patients is projected to grow considerably in the coming years [3]. Some UC patients are prone to developing UC-associated intestinal fibrosis (UC-IF) as a complication of the disease. Studies have reported that the incidence of UC-IF is 11% in patients with chronic and progressive UC [4]. The development of UC-IF has been largely overlooked, yet it has significant clinical consequences, including abnormal colonic motility, anorectal dysfunction, fecal incontinence, and potential progression to intestinal stricture and obstruction, thereby impairing the quality of life of patients [5]. The pathogenesis of UC-IF remains unclear. Most studies suggest that it arises from the interaction between immune cells and stromal cells [6]; and is characterized mainly by fibroblast activation, dysregulation of inflammatory mediators, and massive deposition of the extracellular matrix (ECM) [7]. At present, there are no effective treatments for UC-IF. Therefore, exploring effective therapeutic targets and drugs against UC-IF is highly important.

Neutrophils eliminate microorganisms by releasing antimicrobial peptides and proteases and through phagocytosis. However, when this process fails, neutrophils release nuclear NE and MPO, which together form neutrophil extracellular traps (NETs). These NETs can capture, neutralize, and degrade invading microbes extracellularly via their proteolytic enzymes [8, 9]. Thus, under physiological conditions, NETs play a positive role. However, abnormally high release of NETs can exacerbate inflammation and disrupt tissue structure [10]. NETs are involved in various pathological processes of UC, including releasing cytokines to aggravate inflammation [11]; damaging the intestinal mucosal barrier [12]; triggering pathological coagulation [13]; contributing to biological agent resistance [14], interacting with the intestinal flora [15]; and promoting tumor occurrence, progression, and metastasis through inflammation [16].

Studies have shown that the pathogenesis of fibrosis in multiple organs is associated with the key regulatory role of NETs. For example, NETs promote liver fibrosis progression by driving abnormal collagen accumulation [17]. NETs can also significantly accelerate pulmonary scarring by activating TGF-β1, thus exacerbating pulmonary fibrosis [18]. In addition, the pathogenic role of NETs has also been confirmed in studies on cardiac and renal fibrosis [19, 20]. Therefore, whether NETs are involved in the pathological process of UC-IF, and whether potential NET-related biomarkers and therapeutic targets exist, are the key aspects investigated in this study.

## Materials and methods

### Bioinformatics analysis

#### Dataset collection and processing

**Fig. 1:**
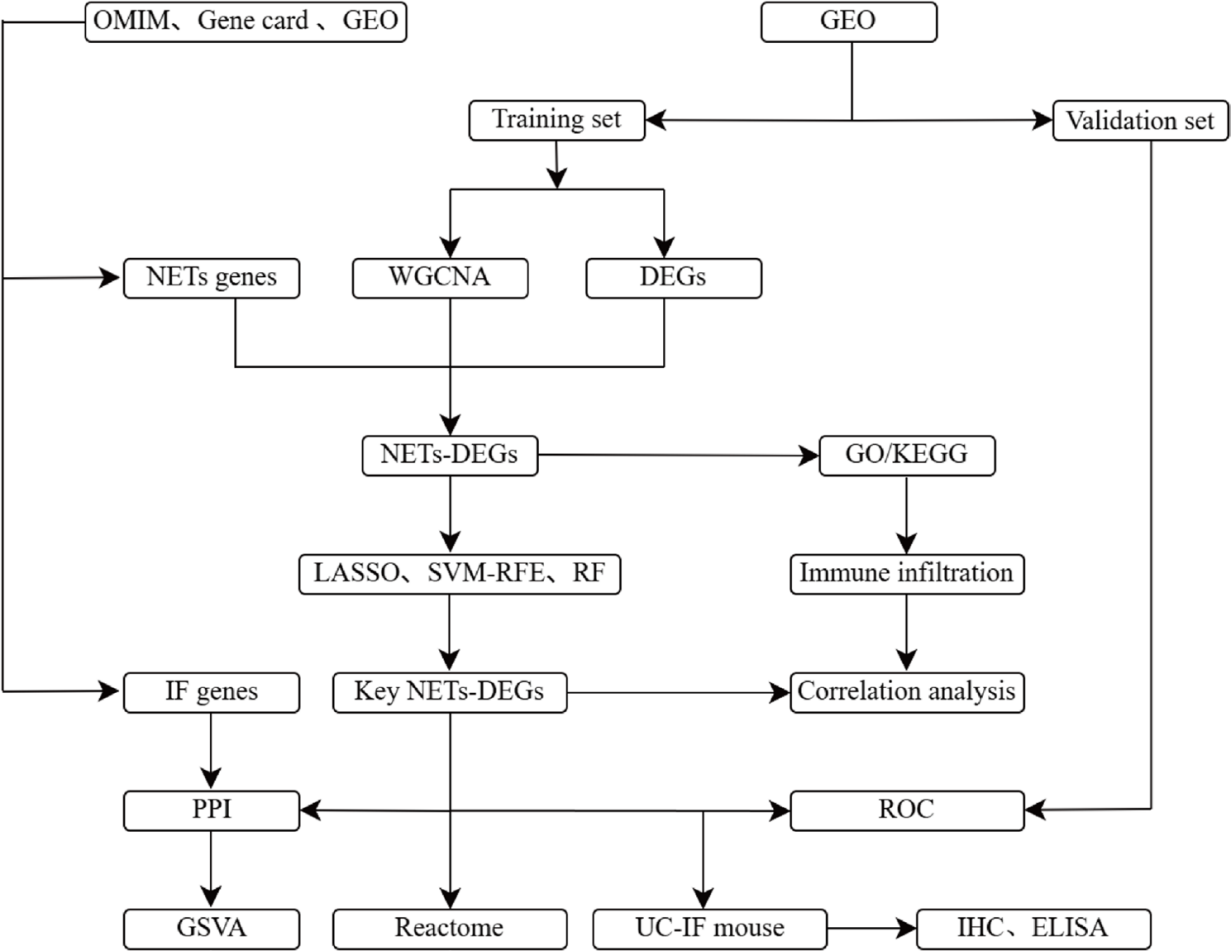
Study flowchart

We obtained 5 UC mRNA datasets (GSE59071, GSE87473, GSE47980, GSE87466 and GSE92415) by searching the Gene Expression Omnibus (GEO) database [21] (https://www.ncbi.nlm.nih.gov/geo/). (Table 1). Data processing was performed via RStudio (version 4.4.3). First, the GSE59071 and GSE87473 datasets were merged via the “*dplyr*” package. Principal component analysis (PCA) was then conducted with the “*stats*” package to evaluate batch differences in the merged dataset. Subsequently, batch variation was corrected with the “*sva*” package. The corrected gene set was finally defined as the training set. NET-related and UC-IF-related genes were obtained from the Online Mendelian Inheritance in Man (OMIM) [22](https://www.omim.org/), GeneCards [23](https://www.genecards.org/), and NCBI Gene databases [21]. After merging and deduplication, gene sets containing 1576 and 2420 genes, respectively were obtained for subsequent analyses (Fig. 1).

**Table 1:** Sample information

GEO Dataset ID	Microarray Platform	RNA Category	Analyzed Samples	Tissue Origin
GSE59071	GPL6244	mRNA	Con: UC = 11:97	Colonic mucosal
GSE87473	GPL13158	mRNA	Con: UC = 21:106	Colonic mucosal
GSE47908	GPL570	mRNA	Con: Left-sided colitis, pancolitis, UC = 15: 45	Colonic mucosal
GSE87466	GPL13158	mRNA	Con: UC = 21:87	Colonic mucosal
GSE92415	GPL13158	mRNA	Con: UC = 21:162	Colonic mucosal

#### Screening of UC-related differentially expressed genes (UC-DEGs)

In this study, the “*limma*” package was employed for normalization of the training set and removal of genes with low expression (expression value < 1). We set the screening thresholds at *p* < 0.05 and |*log₂FC*| > 1 to identify UC-DEGs, and adjusted the P-values for multiple testing. Finally, the top 10 genes with significant differences were displayed, and a heatmap was generated to show the top 50 genes ranked by expression value, via the “*ggplot2*” package.

#### Identification of UC phenotype-related module genes

In this study, WGCNA was performed on the training set via the “*WGCNA*” package to identify the gene modules most strongly associated with UC patient phenotypes. First, excluding outlier samples with a height cutoff of 95 in the sample dendrogram, we constructed a signed network with a soft threshold of *β* = 6. Modules were identified using the unsigned topological overlap matrix (TOM) with the parameters minModuleSize = 50, deepSplit = 1, and subsequently merged at a mergeCutHeight of 0.6. The module most significantly associated with UC was then determined through module-trait correlation analysis. Finally, the “*UpSet*” package was subsequently applied to identify overlapping genes among UC-DEGs, key module genes derived from WGCNA, and NET-related genes. These overlapping genes were defined as NET-related differentially expressed genes (NET-DEGs).

#### Enrichment analysis of NET-DEGs

In this study, GO and KEGG enrichment analyses were performed on NET-DEGs to elucidate the biological functions and signaling pathways involved. The GO analysis encompassed 3 subcategories: biological process (BP), cellular component (CC), and molecular function (MF). Gene ID conversion and enrichment calculations were carried out using the “*clusterProfiler*” package with the human annotation database “*org.Hs.eg.db*”. Terms with an adjusted P-value < 0.05 were considered significantly enriched. The top 5 significantly enriched terms from each of the BP, CC, MF, and KEGG categories were visualized as grouped bar charts using the “*ggplot2*” package.

#### Identification of key NET-DEGs

This study applied 3 independent machine learning methods, including LASSO, SVM-RFE, and RF, to NET-DEGs via the “*glmnet*”, “*e1071*”, and “*randomForest*” packages. LASSO achieves automatic feature selection and model sparsity via L1 regularization, which effectively addresses overfitting and multicollinearity in data with many features [24]. In this study, 10-fold cross-validation was used to select the optimal penalty parameter *λ*, with the minimum mean squared error serving as the model performance criterion. Genes with non-zero regression coefficients were retained as candidates from the LASSO analysis. The SVM-RFE algorithm is suitable for high-dimensional data and obtains robust key genes by recursively removing unimportant features [25]. RF is based on ensemble learning and makes decisions by constructing multiple decision trees. It exhibits strong robustness to high-dimensional data, noise, and multicollinearity, with a low risk of overfitting. It can output gene importance scores, enabling stable and efficient screening of key features [26]. To improve the robustness of the results, important genes identified by each model were defined as key NET-DEGs on the basis of their intersection.

#### Protein-Protein Interaction network (PPI) construction and functional enrichment analysis of key NET-DEGs

In this study, the STRING database [27] (https://cn.string-db.org/) was used to establish a protein-protein interaction (PPI) network, with the aim of evaluating the associations between key NET-DEGs and genes involved in canonical fibrotic pathways. Specifically, the core NET-DEGs and fibrotic pathway-related genes were submitted to STRING with the organism set to *Homo sapiens*, and a combined interaction score threshold of 0.4 was applied. The Key NET-DEGs were subsequently identified based on their interaction relationships, and the PPI network was visualized. Furthermore, pathway enrichment analysis of the finalized key NET-DEGs was performed using the “*ReactomePA*” package to explore the biological pathways in which these genes participate. Terms with an adjusted P-value < 0.05 were considered significantly enriched, and the results were visualized using the “*ggplot2*” package.

#### Gene set variation analysis (GSVA) and correlation analysis of key NET-DEGs

This study conducted gene set variation analysis (GSVA) on the retrieved UC-IF genes using the training set to assess gene set activity in the samples, and visualized the GSVA scores of the Con group and UC group via violin plots. In addition, to explore the associations between the expression of key NET-DEGs and pathway activity, Spearman correlation analysis between key NET-DEGs and GSVA scores was performed via the “*stats*” package. This method is suitable for omics data that do not follow a normal distribution and can robustly evaluate the nonlinear correlations between gene expression and GSVA pathway activity. The results were subsequently visualized.

#### Expression and validation of key NET-DEGs

To further validate the expression stability and clinical utility of the key NET-DEGs, their expression levels were externally validated in the independent cohort. Receiver operating characteristic (ROC) curve analysis was performed to evaluate the ability of each candidate gene to discriminate UC from control samples. Genes with an area under the curve (AUC) greater than 0.7 were considered to have satisfactory discriminatory capacity, supporting their potential as diagnostic biomarkers and therapeutic targets. The results were subsequently visualized.

#### Immune infiltration analysis

In this study, the CIBERSORT deconvolution algorithm was adopted to quantify the relative infiltration abundances of 22 immune cell subtypes in the training dataset. The expression matrix and sample grouping information were imported first; missing values and negative values were eliminated, and cross-sample normalization was conducted via the normalizeBetweenArrays function from the “*limma*” package to remove batch bias. Using the official LM22 signature gene matrix as the reference template, we set permutations = 1000 and quantile normalization (QN = TRUE) to calculate the infiltration proportion of immune cells in each sample. Spearman rank correlation analysis was performed between the immune abundance matrix and the expression levels of key NET-DEGs, and all analytical results were subsequently visualized.

### Animal experiment validation

#### Experimental reagents and instruments

Dextran Sulfate Sodium Salt (36,000–50,000 M.Wt., MP Biomedicals, USA, Cat. No. 0216011080); Hematoxylin-Eosin (HE) High-Definition Constant Staining Kit (Servicebio Technology Co., Ltd., Wuhan, China, Cat. No. G1076); Masson’s trichrome stain kit (Solarbio Science Technology Co., Ltd., Beijing, China, Cat. No. G1340); IL1RA rabbit mAb (Signalway Antibody LLC, USA, Cat. No. 56106); MUC1 rabbit pAb (Signalway Antibody LLC, USA, Cat. No. 53029); SERPINA1 rabbit pAb (Signalway Antibody LLC, USA, Cat. No. 32119); α-SMA rabbit pAb (Signalway Antibody LLC, USA, Cat. No. 41550); Collagen I rabbit pAb (Signalway Antibody LLC, USA, Cat. No. 65024); MPO rabbit pAb (Signalway Antibody LLC, USA, Cat. No. 41742); Histone H3 (Citrulline Arg2 + Arg8+Arg17) Rabbit mAb (Immunoway Biotechnology Co., Ltd., USA, Cat. No. YM9591); HRP-conjugated Goat anti-rabbit IgG (H + L) (Abbkine Scientific Co., Ltd., China, Cat. No. A21020); DyLight 594-conjugated Goat anti-rabbit IgG (H + L) (Abbkine Scientific Co., Ltd., China, Cat. No. A23420); DAPI (Beijing Solarbio Science & Technology Co., Ltd., China, Cat. No. ID22502).

Microplate Reader (AMR-100, Aosheng Instrument Co., Ltd., Hangzhou, China); High-Speed Microcentrifuge (TG16W, Xiangyi Instrument Co., Ltd., Changsha, China); RM2135 Microtome (RM2135, Leica Microsystems, Wetzlar, Germany); BX43 Optical Microscope and UC90 Imaging System (BX43 & UC90, Olympus Corporation, Tokyo, Japan).

#### DSS-induced UC-IF mouse model

Twelve male C57BL/6J mice (20–22 g) were obtained from Liaoning Changsheng Biotechnology Co., Ltd. (Animal License No. SCXK (Liao) 2025-0001). The mice were housed in an SPF barrier facility under constant environmental conditions: temperature of 22 ± 2 °C, relative humidity of 50% ± 10%, and a 12-hour light/12-hour dark photoperiod. After 7 days of acclimatization, the mice were randomly divided into a control group (Con) and a model group (Mod), with 6 mice in each group. The mice in the Mod group were given drinking water containing 2% DSS for 5 days followed by pure water for 7 days as 1 cycle, for a total of 3 cycles. The mice in the Con group received pure water throughout the experiment. During the modeling period, the mice in both groups had free access to food.

#### Specimen collection

After 3 modeling cycles, all the mice were fasted for 12 h with free access to water. Mice were deeply anesthetized with 4% isoflurane in oxygen (flow rate: 1 L/min) for induction. After the loss of righting reflex, blood samples were collected from the retro-orbital venous plexus. Following blood collection, all mice were euthanized by cervical dislocation, and colon tissue samples were isolated. Isoflurane inhalation anesthesia was used to alleviate pain and stress, ensuring animal welfare and compliance with the requirements of the Experimental Ethics Committee of the First Affiliated Hospital of Hebei University of Chinese Medicine. After blood centrifugation, the serum was collected and immediately frozen for storage. Mouse colon tissues were dissected and isolated and fixed with 4% paraformaldehyde (PFA).

#### Hematoxylin-eosin (HE) and Masson’s trichrome staining

Mouse colon tissues were fixed in 4% paraformaldehyde, dehydrated through graded ethanol, cleared with xylene, and embedded in paraffin. The paraffin blocks were cut into 5 μm-thick sections. The sections were deparaffinized by sequential immersion in xylene I and xylene II, and then rehydrated through absolute ethanol I, absolute ethanol II, 95% ethanol, 85% ethanol, and 75% ethanol in descending order, followed by a final rinse with distilled water. The sections were subjected to two separate staining procedures. For HE staining, hematoxylin was used to label cell nuclei, followed by eosin as a cytoplasmic counterstain. For Masson’s trichrome staining, hematoxylin, acid fuchsin and aniline blue staining solutions were applied sequentially. All stained slides were dehydrated in graded ethanol, cleared with xylene, and mounted with neutral balsam. Observations were performed under an optical microscope. HE staining was used to evaluate the severity of pathological lesions, including mucosal inflammatory cell infiltration, crypt structural damage and epithelial erosion, while Masson’s trichrome staining was employed to assess collagen deposition and the degree of colonic fibrosis.

#### Immunohistochemical (IHC) staining

Paraffin-embedded mouse colon tissues were sectioned at a thickness of 5 μm. After deparaffinization in xylene and full rehydration through a graded ethanol series, the sections were subjected to high-pressure antigen retrieval in 3% citrate buffer. Following washing with PBS, hydrogen peroxide solution was applied to block endogenous peroxidase activity, and 4% bovine serum albumin (BSA) was used to block non-specific binding sites. After blocking, the sections were incubated overnight at 4 °C with primary antibodies against IL1RA (rabbit mAb), MUC1 (rabbit pAb), SERPINA1 (rabbit pAb), α-SMA (rabbit pAb) and Collagen I (rabbit pAb) at a uniform dilution of 1: 200. On the next day, the slides were thoroughly washed with PBS, then incubated with HRP-conjugated goat anti-rabbit IgG secondary antibody (1: 2000) at 37 °C for 20 min, followed by another PBS wash. DAB chromogen was added for color development. Nuclei were counterstained with hematoxylin, differentiated in hydrochloric acid-alcohol, and blued under running tap water. After dehydration, clearing and mounting, images were captured under an optical microscope. Quantitative analysis of protein expression was performed using Servicebio Aipathwell image analysis software.

#### Immunofluorescence (IF) staining

Paraffin sections of mouse colon tissues were subjected to deparaffinization, rehydration and antigen retrieval, followed by permeabilization with PBS containing 0.2% Triton X-100 at room temperature for 15 min to improve membrane permeability. After washing with PBS, the sections were blocked with 5% BSA at room temperature for 30 min to eliminate non-specific binding. Subsequently, the sections were incubated overnight at 4 °C in the dark with primary antibodies: Histone H3 (Citrulline Arg2 + Arg8+Arg17) rabbit mAb and MPO rabbit pAb at a dilution of 1:300. On the next day, the slides were washed with PBS and incubated with DyLight 594-conjugated goat anti-rabbit IgG secondary antibody (1:2000) at room temperature in the dark for 1 h. After thorough rinsing with PBS, cell nuclei were counterstained with DAPI working solution by incubation at room temperature away from light for 5 min. Following rinsing, the sections were mounted with anti-fade mounting medium. Fluorescent images were captured under a fluorescence microscope, and quantitative analysis of protein expression was performed using Servicebio Aipathwell image analysis software.

#### Enzyme-Linked Immunosorbent Assay (ELISA)

After serial dilution of the standard solutions, the standards, samples, and matching reagents were loaded into the microplate according to the manufacturer’s instructions. Except for the blank wells, 50 µL of enzyme conjugate reagent was added to each well. The plate was sealed with sealing film and incubated at 37 °C for 30 min. Subsequently, all wells were washed with washing buffer, followed by the addition of chromogenic substrate. The plate was incubated at 37 °C in the dark for 10 min to allow color development. The reaction was terminated by adding stop solution, and the absorbance (OD value) of each well was measured at 450 nm.

#### Statistical analysis

This study performed statistical analysis and plotting via RStudio 4.4.3 and GraphPad Prism 11.0. For comparisons between animal groups, we used the independent sample t-test when the data were normally distributed and had equal variances; when variances were unequal, we used Welch’s t-test; and when the data were not normally distributed, we used the Mann-Whitney U test. Normally distributed data were expressed as mean ± standard deviation (SD), while non-normally distributed data were presented as median (interquartile range). A *p* < 0.05 was considered significant.

## Results

### Bioinformatics results

#### Identification of UC-DEGs

**Fig. 2:**
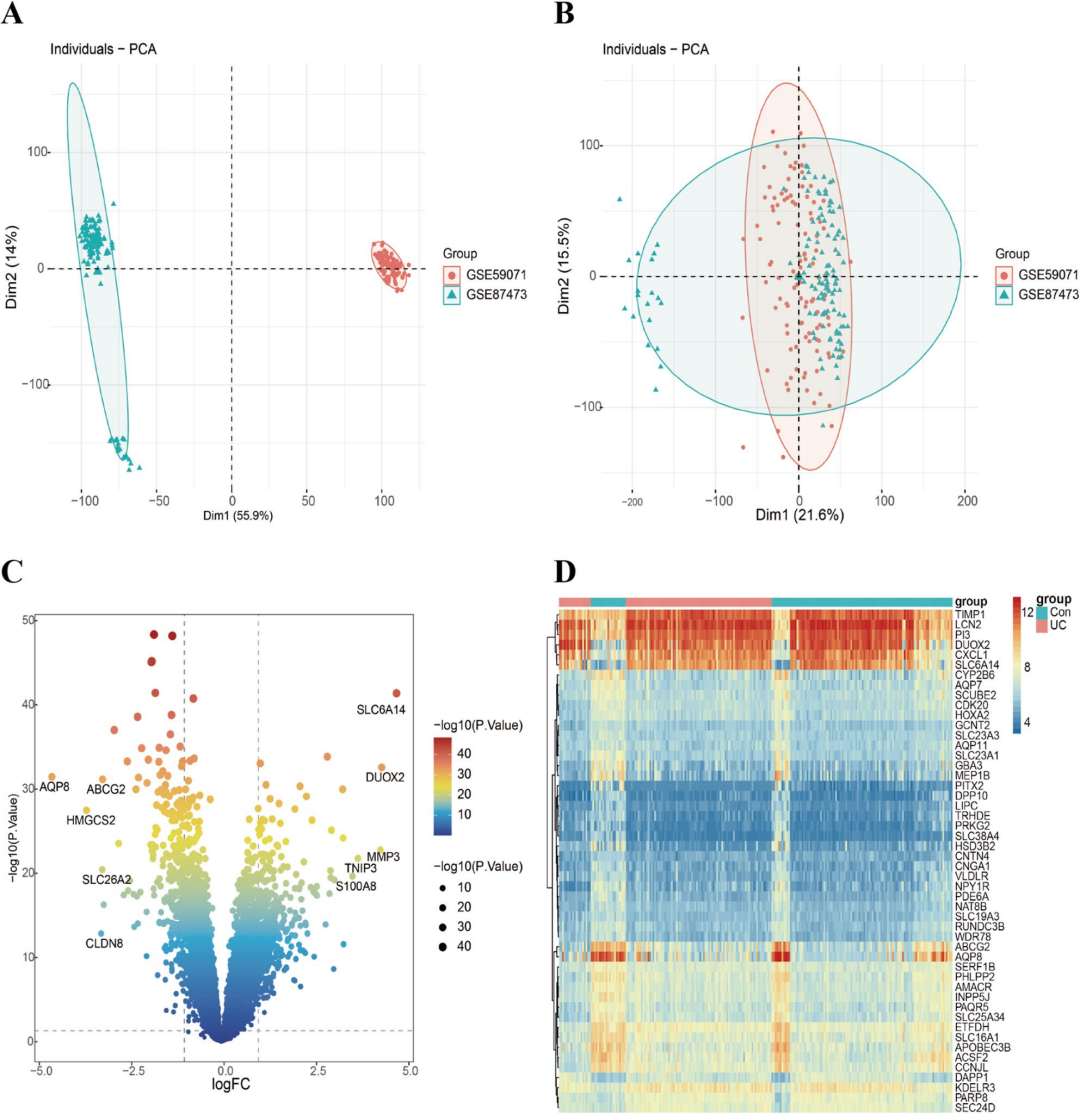
PCA and identification of UC-DEGs in UC. **A** PCA plot of the training set before correction. **B** PCA plot of the training set after correction. **C** Volcano plot of UC-DEGs in the training set. **D** Heatmap of the top 50 UC-DEGs

After performing the principal component analysis, we obtained the combined training set of the GSE59071 and GSE87473 datasets (Fig. 2A-B). According to the screening criteria described in the Methods section, we performed differential expression analysis on the training set. This analysis yielded 641 DEGs, including 385 upregulated genes and 256 downregulated genes. A volcano plot of differential expression was generated, and the top 50 DEGs with the highest expression levels were visualized as a heatmap (Fig. 2C-D).

#### Identification of UC module genes and NET-DEGs

**Fig. 3:**
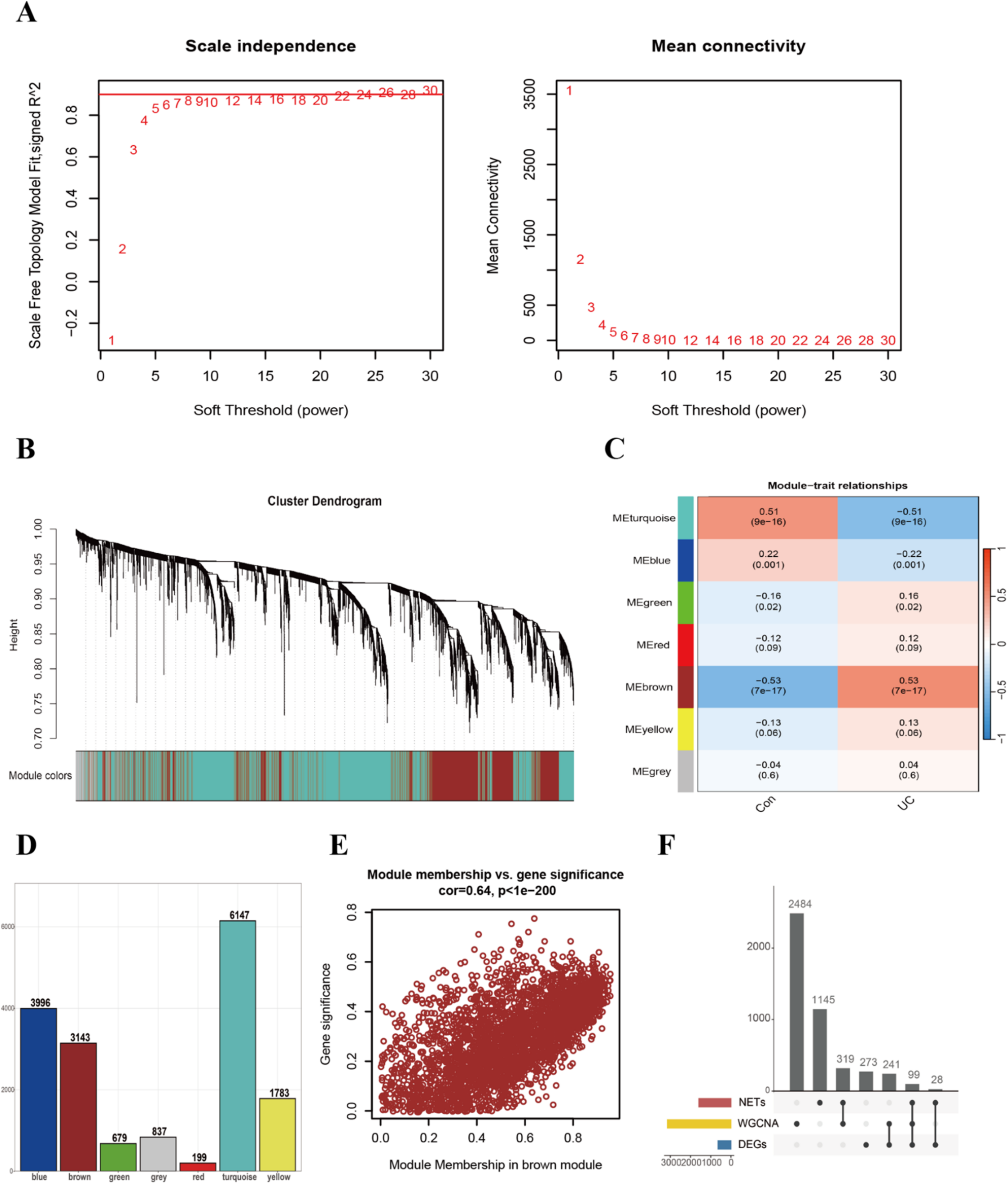
WGCNA expression modules. **A** The optimal soft-thresholding power recommended by the algorithm is 6. **B** Gene clustering dendrogram. **C** Heatmap of the correlations between different module genes and UC. **D** Bar plot of the number of genes in each module. **E** Scatter plot of module gene correlations. **F** UpSet plot showing the intersection of 3 gene sets

We performed WGCNA on the training set. On the basis of the selected soft-thresholding power, we obtained 7 modules, among which the genes in the brown module presented the most significant positive correlation with the UC phenotype (*cor* = 0.64, *p* < 1e-200). This module contains 1,633 genes (Figs. 3A-E). Finally, we performed cross-identification of the UC-DEGs in the training set, identified the genes of the module with the most significant positive correlation with the UC phenotype from the WGCNA, retrieved NET-related genes, and finally obtained 99 NET-DEGs (Fig. 3F).

#### Results of enrichment analysis of NET-DEGs

**Fig. 4:**
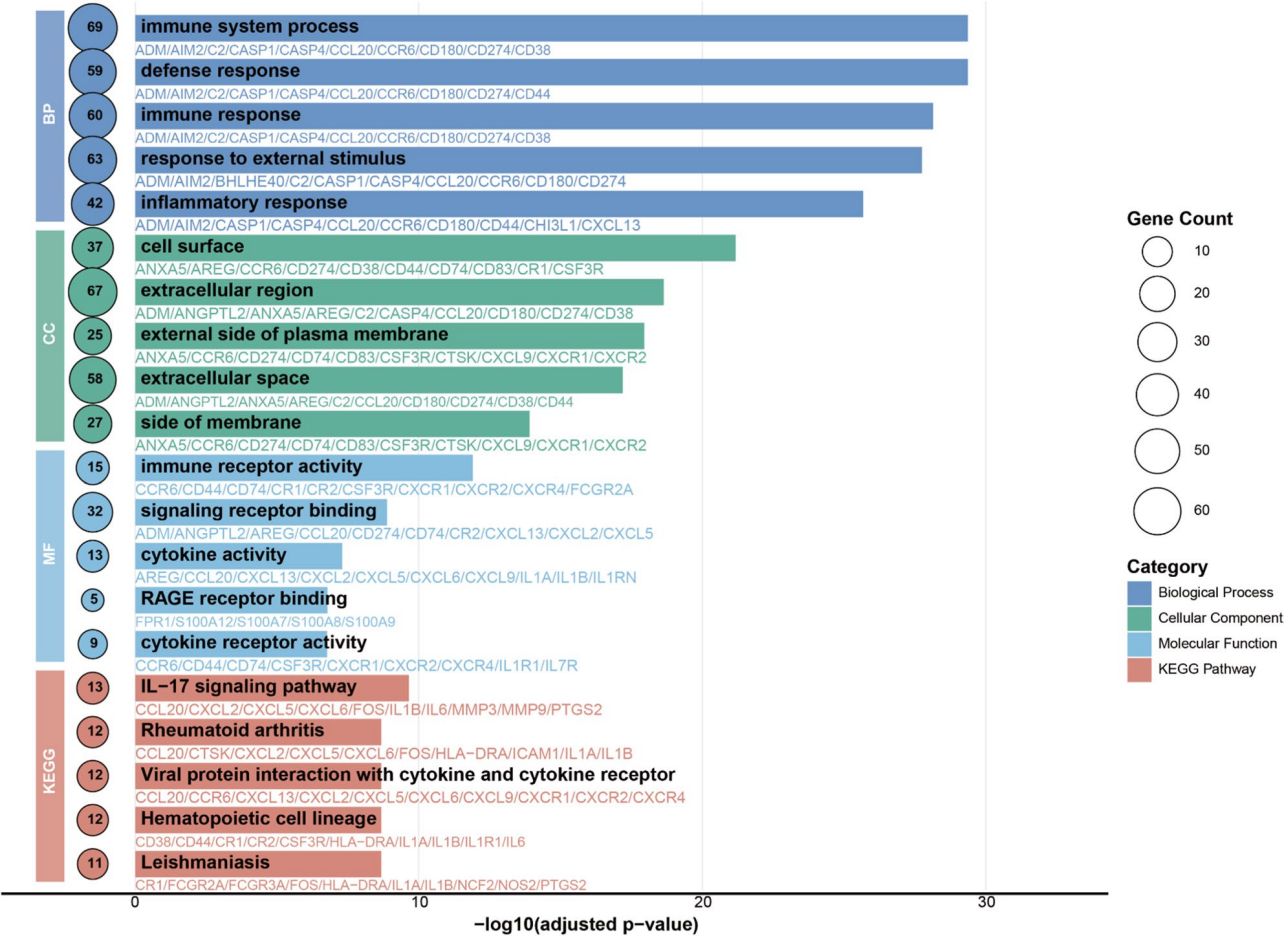
Bar chart of the GO and KEGG results for NET-DEGs

The molecular functions of the NET-DEGs involved primarily immune receptor activity, signaling receptor activity, cytokine activity, RAGE receptor binding, and cytokine receptor activity. These genes were associated mainly with cellular components such as the cell surface, membrane region, and external side of the plasma membrane. The enriched biological processes were mainly associated with immune processes, the immune response, and the defense response. KEGG analysis revealed that the NET-DEGs were involved mainly in the following pathways: cytokine-cytokine receptor interaction, rheumatoid arthritis, viral protein interaction with cytokines and cytokine receptors, the IL-17 signaling pathway, and leishmaniasis (Fig. 4).

#### Results of machine learning

**Fig. 5:**
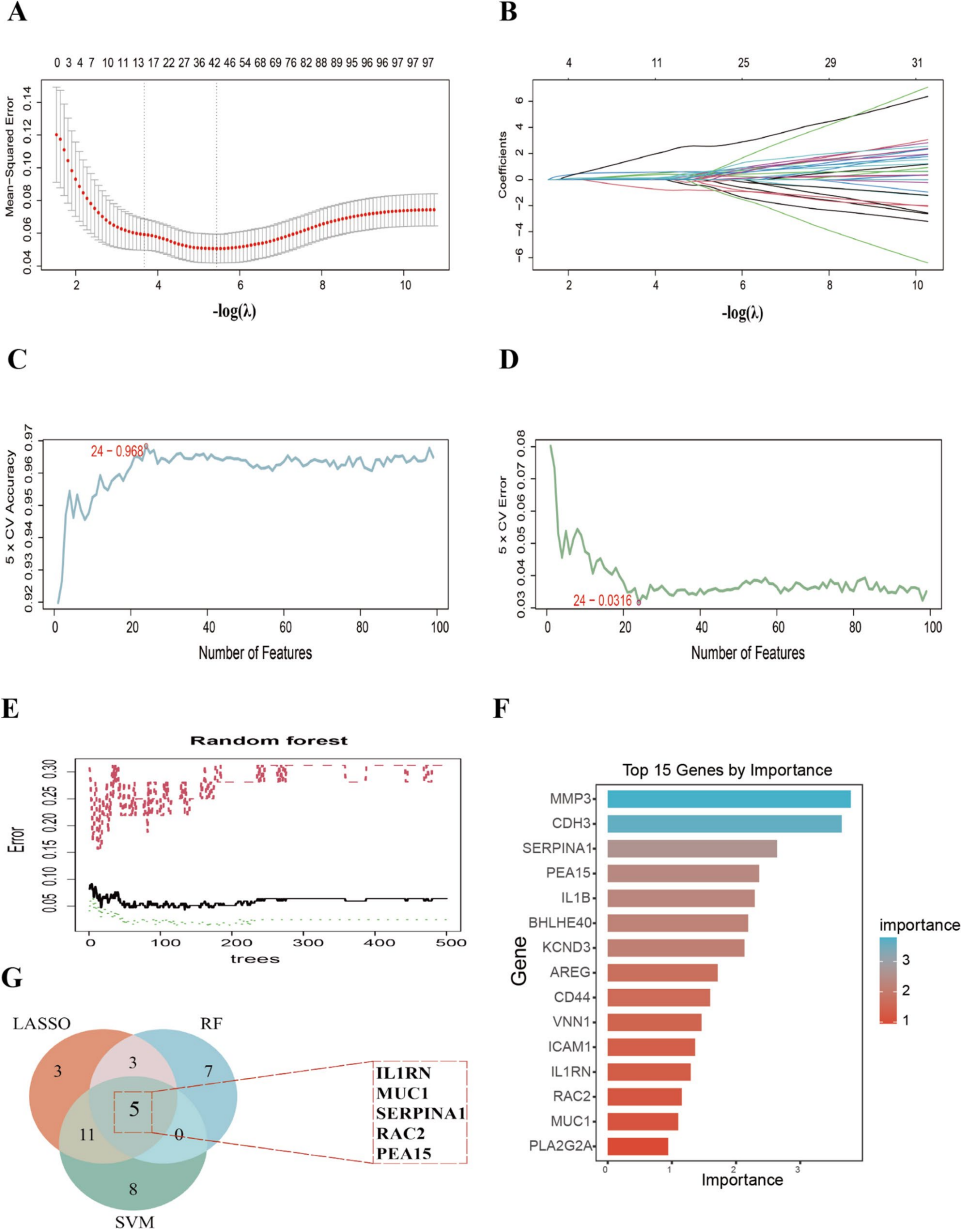
Three machine learning models for identifying NET-DEGs. **A**-**B** Analysis via the least absolute shrinkage and selection operator (LASSO) algorithm. **C**-**D** Analysis via the support vector machine (SVM) algorithm. **E**-**F** Analysis via the random forest (**R**-**F**) algorithm. **G** Venn diagram showing the overlap of the 3 models

We constructed 3 machine learning models to reduce algorithm-specific bias. Using the LASSO regression model, we identified 22 candidate genes (Fig. 5A-B). The SVM-RFE model identified 24 candidate genes, with an accuracy of 96.8% (error rate: 3.16%) (Fig. 5C-D). The random forest model identified 15 candidate genes (Fig. 5E-F). To improve robustness, we took the intersection of the important genes from the 3 models via a Venn diagram, which yielded 5 overlapping genes: IL1RN, MUC1, SERPINA1, RAC2, and PEA15. Therefore, these genes were identified as the key NET-DEGs (Fig. 5G).

#### Construction of the PPI network and enrichment analysis results of key NET-DEGs

**Fig. 6:**
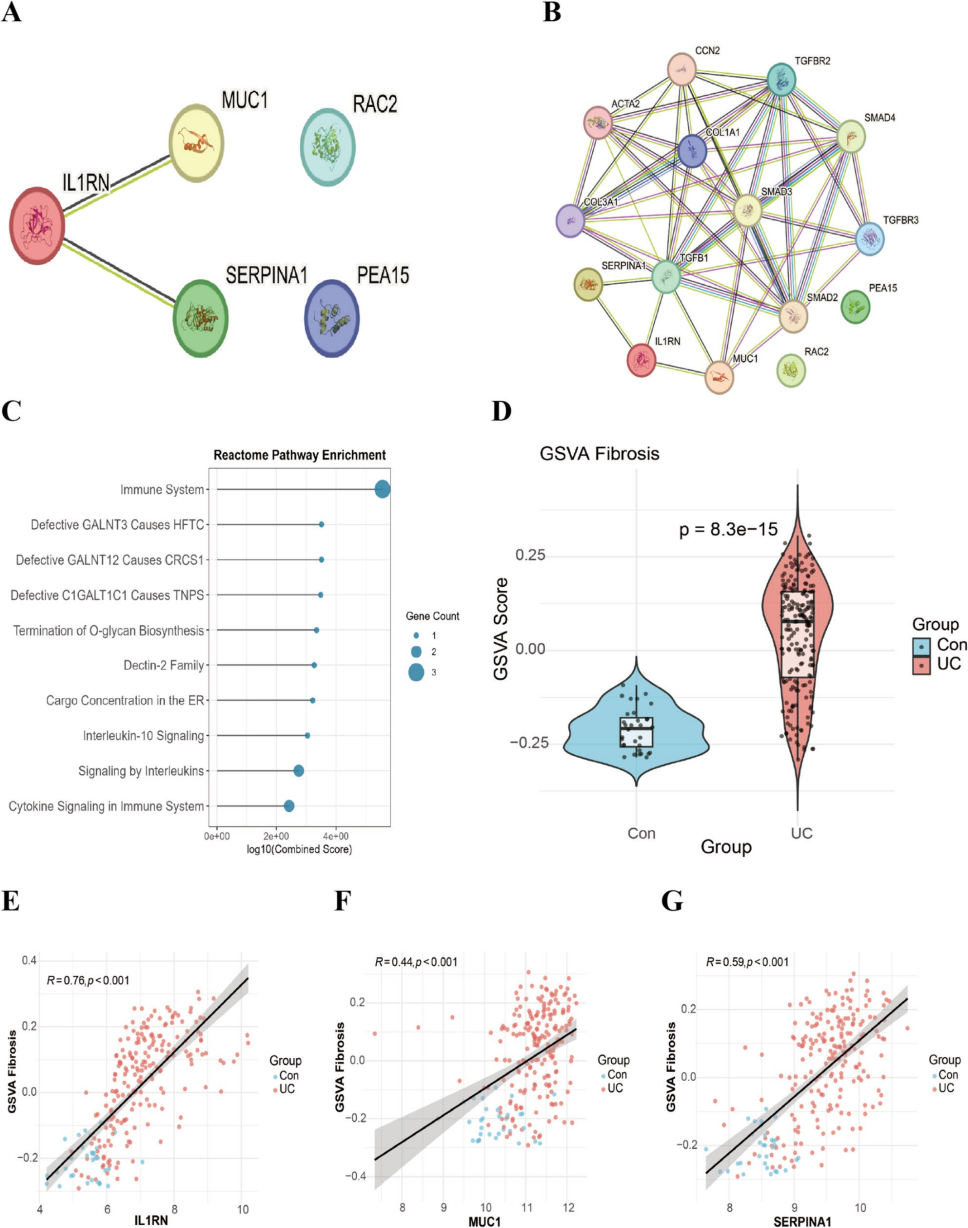
PPI network and GSVA analysis. **A** PPI network diagram of key NET-DEGs. **B** PPI network diagram of NET-DEGs and fibrosis pathway genes. **C** Lollipop plot of enrichment analysis of key NET-DEGs. **D** Violin plot of GSVA scores. **E**-**G** Scatter plots of correlation between key NET-DEGs and GSVA scores

According to the PPI network, we found that IL1RN could interact with MUC1 and SERPINA1 (Fig. 6A). To explore whether these key genes are involved in the UC-IF process, we retrieved key genes of classic fibrotic pathways from the literature, including TGFB1, TGFBR1, SMAD2, and SMAD3, as well as the collagen synthesis genes COL1A1 and CCN2 [28–31]. We observed that IL1RN, MUC1, and SERPINA1 could interact with these fibrosis pathway genes. These findings suggest that these genes may activate the classic TGF-β pathway, thereby promoting fibroblast activation and contributing to UC-IF progression. Therefore, we identified IL1RN, MUC1, and SERPINA1 as key NET-DEGs (Fig. 6B). Meanwhile, we performed Reactome enrichment analysis on the key NET-DEGs. The results revealed that IL1RN, MUC1, and SERPINA1 were enriched mainly in the immune response, interleukin signaling pathway, O-glycosylation biosynthesis, and mucosal barrier-related pathways. These findings indicate that these 3 genes are involved in the core pathological processes of UC-IF, including the regulation of inflammation, disruption of the mucosal barrier, and extracellular matrix (ECM) remodeling (Fig. 6C).

#### Results of GSVA and correlation analysis of key NET-DEGs

We performed GSVA scoring on the retrieved UC-IF-related genes from the training set, and the results revealed that the GSVA scores of the UC group were significantly greater than those of the Con group, indicating greater activity of fibrosis-related genes in the UC group (Fig. 6D). To further explore the correlations between the expression levels of IL1RN, MUC1, and SERPINA1 and pathway activity, we performed Spearman correlation analysis. These results indicate that the key genes are significantly positively correlated with the following GSVA scores: IL1RN (Spearman *R* = 0.76, *p* < 0.001), MUC1 (Spearman *R* = 0.44, *p* < 0.001), and SERPINA1 (Spearman *R* = 0.59, *p* < 0.001), suggesting that the key genes are closely related to the activity of fibrosis-related pathways (Fig. 6E-G).

#### Expression and validation results of key NET-DEGs

**Fig. 7:**
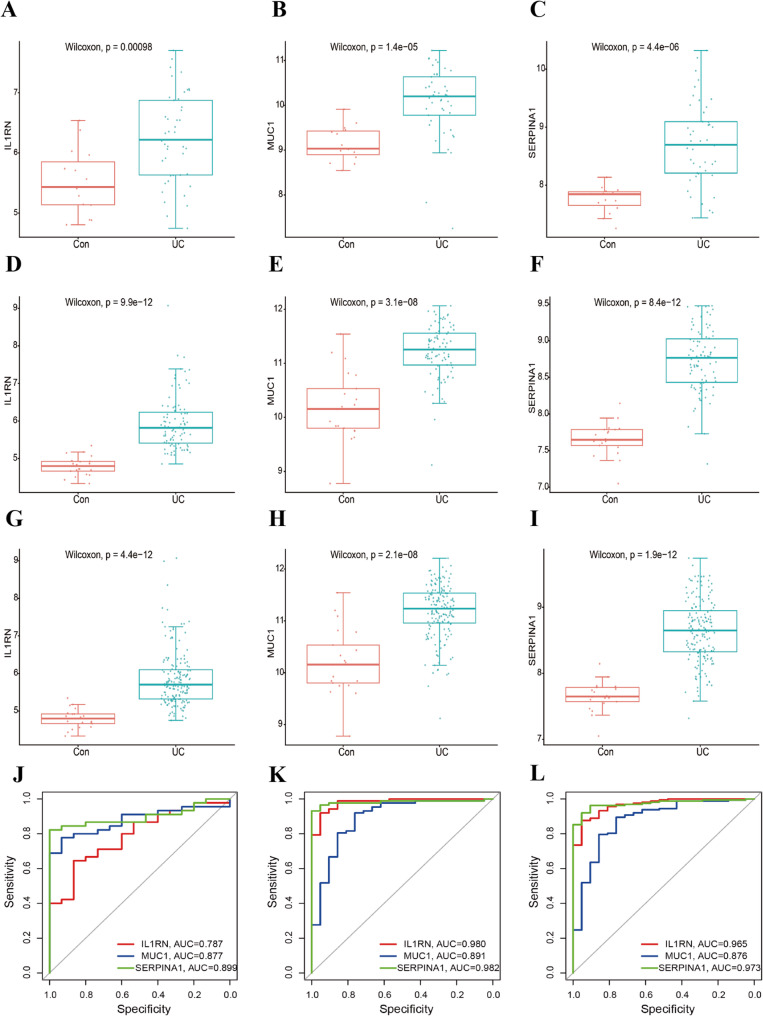
Expression and validation of key NET DEGs. **A**-**C** Expression of key NET DEGs in the GSE47908 dataset. **D**-**F** Expression of key NET DEGs in the GSE87466 dataset. **G**-**I** Expression of key NET DEGs in the GSE92415 dataset. **J** ROC model of key NET DEGs in the GSE47908 dataset. **K** ROC model of key NET DEGs in the GSE87466 dataset. **L** ROC model of key NET DEGs in the GSE92415 dataset

To validate the robustness of IL1RN, MUC1, and SERPINA1, we performed the Wilcoxon rank-sum test (Mann-Whitney U test) across multiple validation cohorts. The results were as follows: GSE47908 (IL1RN: *p* = 0.00098, MUC1: *p* = 1.4e-05, SERPINA1: *p* = 4.4e-06) (Fig. 7A-C), GSE87466 (IL1RN: *p* = 9.9e-12, MUC1: *p* = 3.1e-08, SERPINA1: *p* = 8.4e-12) (Fig. 7D-F), and GSE92415 (IL1RN: *p* = 4.4e-12, MUC1: *p* = 2.1e-08, SERPINA1: *p* = 1.9e-12) (Fig. 7G-I). The expression of these 3 genes in the validation set was consistent with that in the training set, and their expression in the UC group was significantly higher than that in the Con group. Furthermore, receiver operating characteristic (ROC) curves were constructed to evaluate the clinical diagnostic efficacy of the candidate genes in the validation cohorts. The AUC values of the 3 genes in different datasets were as follows: GSE47908 (IL1RN: *AUC* = 0.787, MUC1: *AUC* = 0.877, SERPINA1: *AUC* = 0.899) (Fig. 7J), GSE87466 (IL1RN: *AUC* = 0.980, MUC1: *AUC* = 0.891, SERPINA1: *AUC* = 0.982) (Fig. 7K), and GSE92415 (IL1RN: *AUC* = 0.965, MUC1: *AUC* = 0.876, SERPINA1: *AUC* = 0.973) (Fig. 7L). All genes achieved AUC values greater than 0.7 across all validation datasets, demonstrating excellent and stable diagnostic potential for UC.

#### Results of immune infiltration analysis

**Fig. 8:**
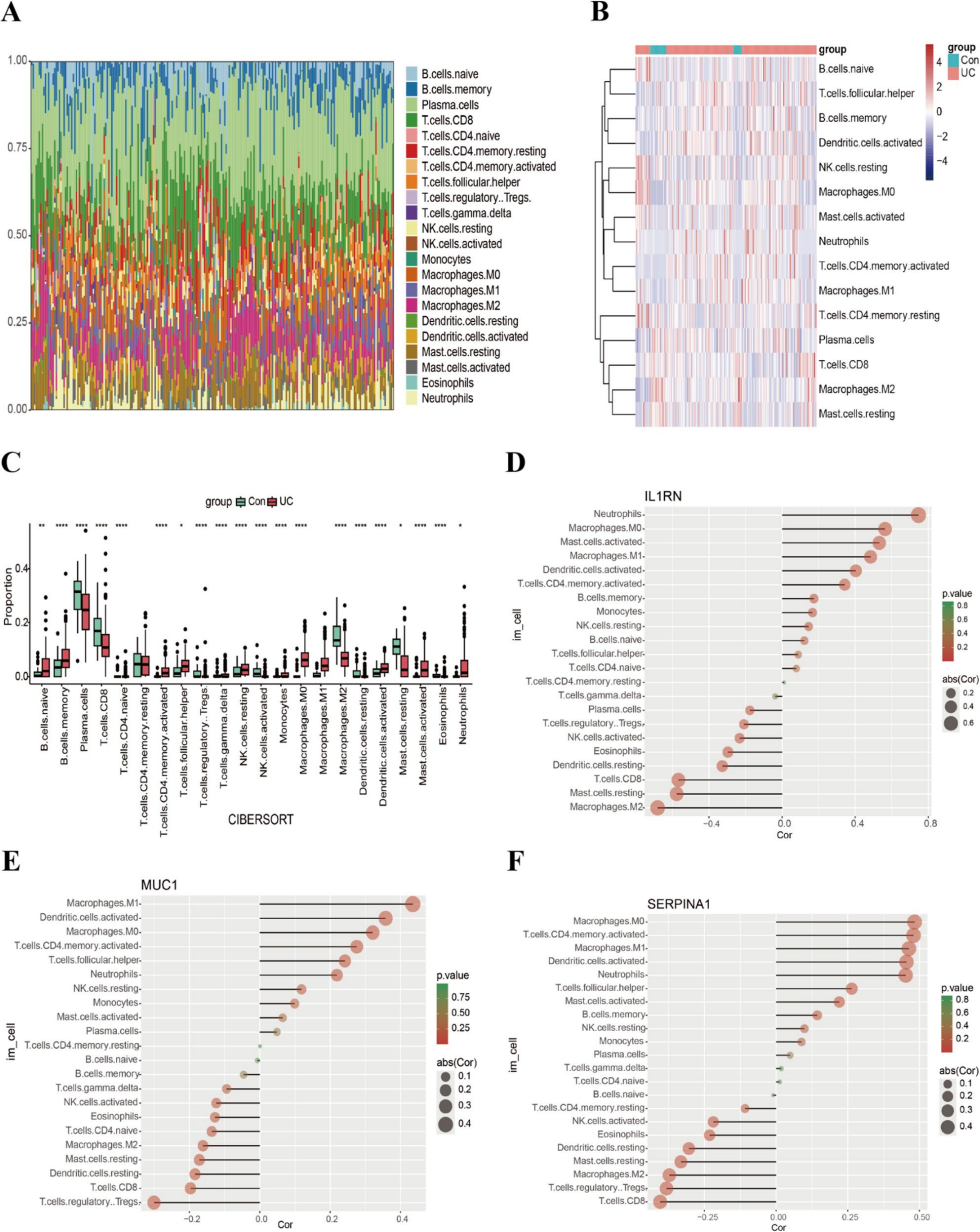
Immune infiltration analysis. **A** Proportion of 22 immune cell types. **B** Clustered heatmap of immune cells. **C** Box plots of immune cells in the Con and UC groups. **D**-**F** Lollipop plots of correlations between key NET-DEGs and immune cells. (*p < 0.05; **p < 0.01; ***p < 0.001; ****p < 0.0001)

We analyzed the distribution and clustering patterns of distinct immune cell types in the training set, and the results indicated that these immune cells may exert vital effects on the progression of UC (Fig. 8A-B). Moreover, we compared the immune cell abundances between the Con group and the UC group. Compared with the Con group, the UC group presented significantly higher abundances of naïve B cells, memory B cells, helper T cells, macrophages (M0, M1), dendritic cells, and neutrophils, and significantly lower abundances of plasma cells, CD8⁺ T cells, and M2 macrophages(Fig. 8C). Next, we analyzed the correlations between key NET-DEGs and immune cells. The results revealed that key NET-DEGs were positively correlated with neutrophils, macrophages (M0, M1), dendritic cells, and activated CD4 memory T cells, and negatively correlated with M2 macrophages, Treg cells, and CD8⁺ T cells, which also confirmed the results obtained above (Fig. 8D-F). These results suggest that key NET-DEGs may mediate immune dysregulation in UC, thereby contributing to the progression of UC-IF.

### Results of in vivo animal validation

#### Results of colon length, HE and Masson’s trichome staining

**Fig. 9:**
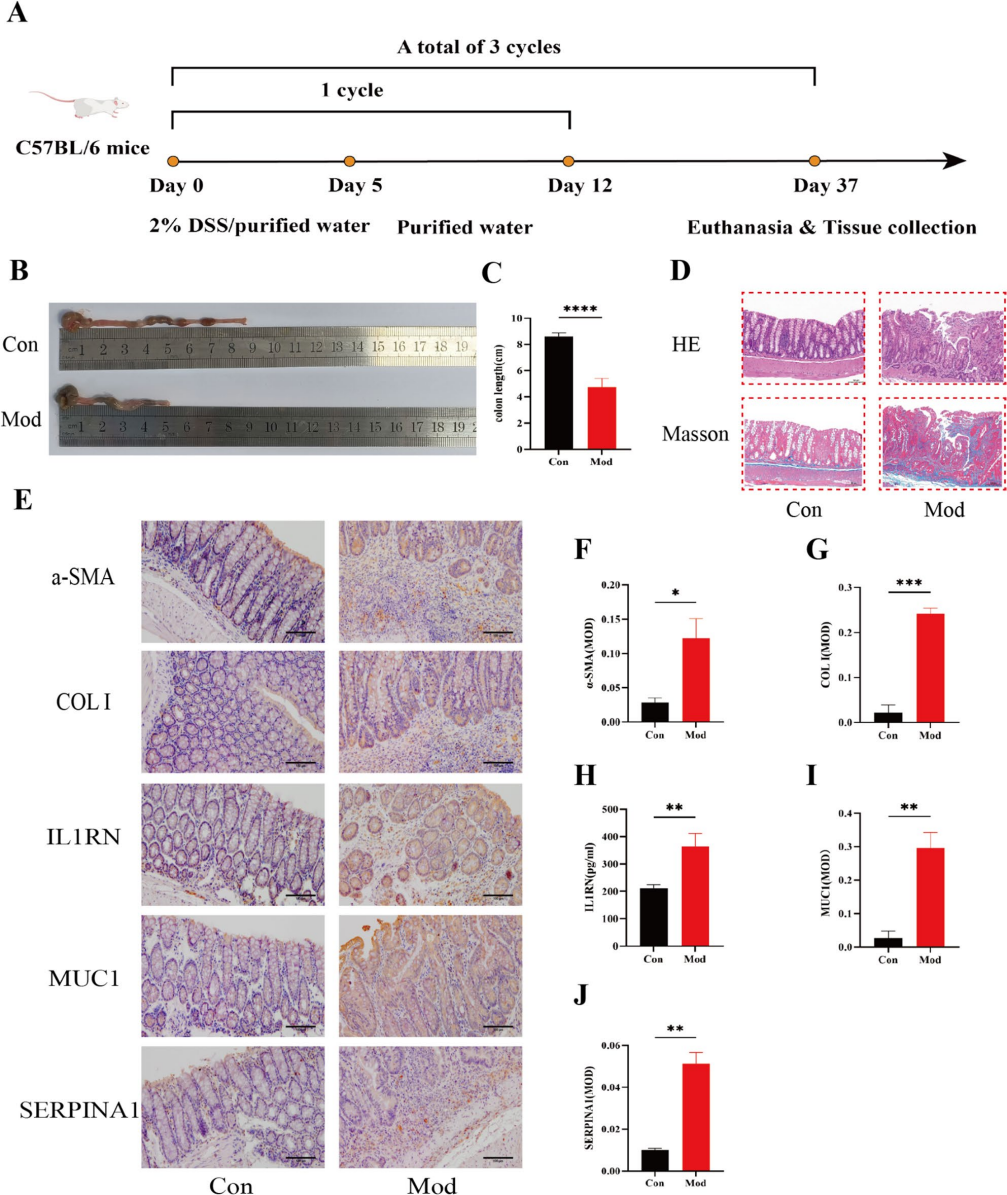
Establishment of the animal model. **A** Flowchart of the mouse modeling cycle. **B**-**C** Changes in colon length between the Con and Mod groups (*n* = 5). **D** HE and Masson staining of mouse colons from the Con and Mod groups. **E**-**J** IHC staining of α-SMA, COL I, IL1RN, MUC1, and SERPINA1 in the colons of mice from the Con and Mod groups (*n* = 3). (*p < 0.05, **p < 0.01, ***p < 0.001, ****p < 0.0001)

We constructed a UC-IF mouse model using 2% DSS (Fig. 9A). Compared with those in the Con group, the colon lengths of the mice in the Mod group were significantly shorter (*p* < 0.0001) (Fig. 9B-C).

HE staining revealed that in the Con group, the colonic mucosa was lined with neatly arranged epithelial cells, with abundant goblet cells, regular crypt morphology, and no obvious inflammatory cell infiltration. In contrast, the Mod group presented discontinuous mucosal surface epithelium with visible erosion and defects. The crypt structure was disorganized or even lost, and the number of goblet cells was markedly reduced. Moreover, extensive infiltration of inflammatory cells, including lymphocytes and neutrophils, was observed in the lamina propria and submucosa (Fig. 9D).

Masson’s trichrome staining revealed a small number of thin blue collagen fibers in the submucosa and muscular layer in the Con group. These fibers were evenly distributed with clear layering, and no abnormal deposition or thickening was detected. In contrast, the Mod group presented abundant dense blue collagen fibers in the submucosa, lamina propria, and muscular layer. These fibers were disorganized and significantly thickened, suggesting excessive collagen deposition and fibrosis (Fig. 9D).

#### Results of immunohistochemistry (IHC) staining

IHC staining and quantitative analysis were performed for the fibrotic markers α-SMA and COL I. The results revealed that the expression levels of α-SMA and COL I were significantly greater in the Mod group than in the Con group (*p* < 0.05, *p* < 0.001), suggesting the activation of colonic fibroblasts in the mice and the successful establishment of the UC-IF mouse model. In addition, we compared the expression of key NET-DEGs between the two groups. Consistent with previous results, the expression levels of IL1RN, MUC1, and SERPINA1 were significantly upregulated in the Mod group relative to those in the Con group (*p* < 0.01, *p* < 0.0001, and *p* < 0.01, respectively) (Fig. 9E-J).

#### Results of immunofluorescence (IF) staining

**Fig. 10:**
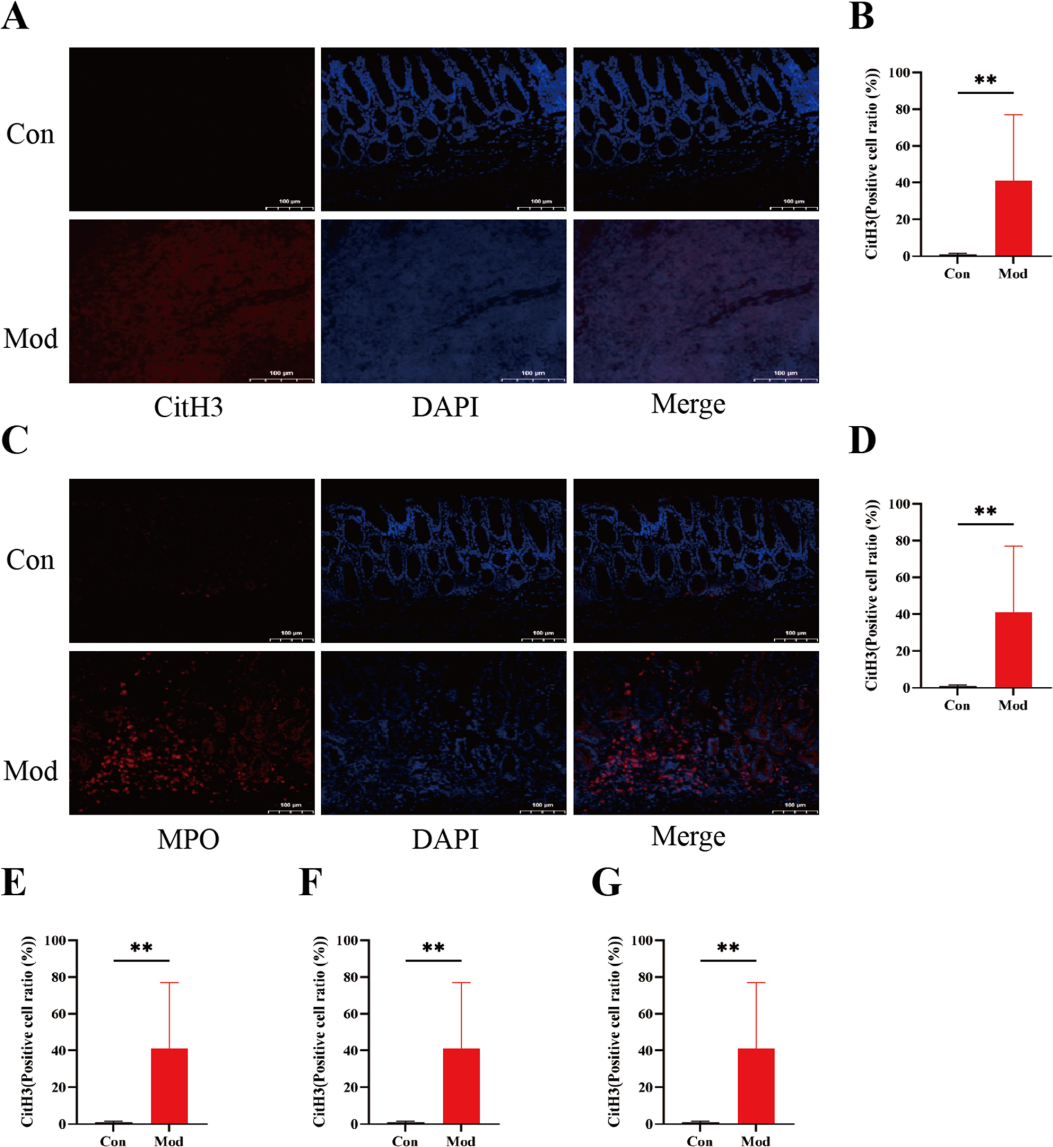
IF staining and ELISA in mice. **A**-**D** IF staining of CitH3 and MPO in the colons of the mice in the Con and Mod groups (*n* = 3). **E**-**G** Quantitative ELISA analysis of serum IL-1RN, MUC1, and SERPINA1 in mice from the Con and Mod groups (*n* = 5). (*p < 0.05, **p < 0.01, ***p < 0.001, ****p < 0.0001)

We verified whether NETs are involved in UC fibrosis by detecting NET markers (CitH3 and MPO) via IF staining. Compared with the Con group, the expression levels of CitH3 and MPO in the Mod group were significantly higher (both *p* < 0.01), suggesting that NETs are involved in UC fibrosis (Fig. 10A-D).

#### Results of ELISA

The serum levels of key NET-DEGs in UC-IF model mice were detected via ELISA. Compared with those in the Con group, the expression levels of IL1RN, MUC1, and SERPINA1 in the Mod group were significantly greater (*p* < 0.01, *p* < 0.0001 and *p* < 0.0001) (Fig. 10E-G).

## Discussion

Intestinal fibrosis is one of the most challenging complications of IBD. Unlike Crohn’s disease (CD), which affects the entire intestinal wall, UC-IF is mainly confined to the mucosal and submucosal layers [32]. Although the incidence of UC-IF is much lower than that of CD, evidence suggests that in patients with long-standing UC, submucosal thickening and remodeling of the muscularis mucosae may also occur [33]. During the course of UC, repeated mucosal epithelial damage and inflammatory cell infiltration activate fibroblasts in the lamina propria and induce their transformation into myofibroblasts. This process upregulates key pro-fibrotic signaling pathways, including the TGF-β1, IL-1, IL-13, and TNF pathways, promoting excessive deposition of ECM components such as collagen and ultimately driving the progression of intestinal fibrosis [34]. As a key pathogenic factor, NETs have become a research hotspot in disease mechanisms in recent years [35]. Studies have confirmed that the expression of the NET-related marker PAD4 is significantly increased in colorectal biopsy samples from patients with UC and CD [36]. In mouse models, the application of deoxyribonuclease I (DNase I) can reduce the release of NETs and alleviate intestinal inflammation as well as the progression of colitis-associated tumors [37]. Similarly, in CD-associated fibrosis, NETs interact with fibroblasts to mediate the fibrotic process [38]. Notably, this study confirmed that NETs are involved in the fibrotic pathological process of UC-IF.

First, this study screened NET-DEGs. Through enrichment analysis, the results revealed that these genes are involved mainly in immune and defense responses, are located primarily in the cell membrane and its outer regions, perform functions such as receptor and cytokine activity, and are enriched in signaling pathways, including cytokine-cytokine receptor interactions and the IL-17 signaling pathway. These findings suggest that NET-DEGs may participate in the pathological processes of UC-related intestinal inflammation and intestinal fibrosis by regulating NET-mediated immune responses. NETs are not only important components of anti-infective immunity, but their abnormal activation can also trigger excessive immune responses, disrupt immune homeostasis, and thereby mediate pathological processes such as inflammation and fibrosis [39]. To further screen the key genes that may play a major role, 3 machine learning models, namely LASSO, SVM-RFE, and RF, were constructed in this study, and IL1RN, MUC1, SERPINA1, RAC2, and PEA15 were identified as key NET-DEGs through overlap analysis. Moreover, we selected key genes in the fibrotic pathway, such as TGFB1, TGFBR1, and COL1A1, which are key components of the TGF-β pathway. We initially reported that IL1RN, MUC1, and SERPINA1 can interact with key genes in the fibrotic pathway, suggesting that these genes may participate in UC-IF by activating the canonical TGF-β pathway and stimulating fibroblasts. Finally, we identified IL1RN, MUC1, and SERPINA1 as key NET-DEGs. Through enrichment analysis, we found that IL1RN, MUC1, and SERPINA1 are enriched mainly in the immune response, interleukin signaling, O-glycosylation biosynthesis, and mucosal barrier-related pathways, indicating that these 3 genes are involved in the core pathological process of UC-IF, which also confirms our speculation.

To test this hypothesis, we downloaded the UC-IF-related gene set from the GeneCards database and performed GSVA scoring on the training set to evaluate its activity across samples. The results confirmed the increased activity of UC-IF genes in the UC group. Notably, IL1RN, MUC1, and SERPINA1 were significantly positively correlated with fibrosis-related pathways, indicating their involvement in UC-IF through these pathways. To further assess the robustness of these genes, we validated them via an independent validation set and performed ROC curve analysis. The results revealed that IL1RN, MUC1, and SERPINA1 were robust in the validation set and exhibited favorable diagnostic and therapeutic potential. These findings strongly support their potential as diagnostic and therapeutic targets for UC-IF.

We used the CIBERSORT algorithm to perform immune infiltration analysis on the training set samples. This revealed both the relative proportions of various immune cell subsets and significantly increased infiltration levels of multiple immune cell types in the UC group. These findings further confirm that UC is characterized by significant immune dysregulation [40]. Moreover, correlation analysis revealed that IL1RN, MUC1, and SERPINA1 were significantly positively correlated with neutrophils, M0 macrophages, M1 macrophages, CD4⁺ T cells, and resting dendritic cells, suggesting that these key genes may cooperate with these immune cells to drive immune dysregulation and contribute to UC-IF progression.

IL1RN is an important inflammatory regulatory factor that competitively binds to interleukin-1 receptors and helps block the proinflammatory and profibrotic effects of IL-1 [41]. The expression of IL1RN is increased in UC, which reduces the production of IL-1β and forms a negative feedback loop to control intestinal mucosal inflammation [42]. However, studies have shown that the expression level of IL1RN is associated with the disease severity of UC, suggesting that genetic variations may impair the function of IL1RN. As a key NET-related gene, it participates in the pathogenesis of UC by regulating NETs [43]. Studies have confirmed that IL1RN can significantly inhibit fibroblast activation and collagen deposition, thereby exerting an anti-hepatic fibrosis effect [44]. These findings provide an important theoretical basis for the involvement of IL1RN in the regulation of UC-IF in the present study.

MUC1 is the main component of mucus secreted by goblet cells, and this mucus forms the intestinal mucus barrier, thereby exerting a protective effect on the intestine [45]. In UC, MUC1 expression is increased, and MUC1 mRNA expression is positively correlated with disease severity [46, 47]. Currently, few studies have directly investigated the regulation of NETs by MUC1, but MUC1 can inhibit the TLR2/3/5 signaling pathway and reduce the secretion of pro-inflammatory factors such as TNF-α and IL-8, which are important inducers of NETs [48]. These findings indirectly suggest that MUC1 plays a regulatory role in NET formation. Moreover, studies have shown that MUC1 overexpression is associated with intestinal inflammation and fibrosis in CD [49], suggesting that MUC1 may serve as a predictive marker of intestinal fibrosis.

SERPINA1 is a plasma protein that participates in various physiological processes such as blood coagulation, inflammation, and the immune response [50]. It is a natural inhibitor of NE that can inhibit NE activity during NET release and negatively regulate the release of NETs, thereby reducing NET-mediated tissue damage and fibrosis [51]. SERPINA1 can serve as an early diagnostic indicator for both UC and colorectal cancer [52, 53]. To validate the bioinformatics findings, a UC-IF mouse model was established using DSS, and the results were further confirmed. Therefore, we conclude that IL1RN, MUC1, and SERPINA1 are reliable NET-related biomarkers for UC-IF.

In summary, IL1RN, MUC1, and SERPINA1 are involved in UC-IF. These genes may serve as potential NET-related biomarkers for UC-IF and merit further investigation. However, this study has several limitations. First, all the data were derived from public databases, which may have batch effects, limited sample sizes, and ethnic and sex biases. Second, we verified the expression of only IL1RN, MUC1, SERPINA1, CitH3, and MPO in the intestinal mucosa by IHC and IF; co-localization experiments were due to funding constraints, so direct evidence of their co-localization is lacking. Given these limitations, more comprehensive studies are needed in the future to further clarify the diagnostic value and clinical potential of the above markers in UC-IF.

## Conclusion

This study confirmed that IL1RN, MUC1, and SERPINA1 are NET-related biomarkers in the process of UC-IF. These biomarkers have good diagnostic efficacy and potential translational value, and are expected to provide new insights and strategies for the early diagnosis, targeted intervention, and clinical management of UC-IF.

## Supplementary Information

Supplementary Material 1: Table S1. NETs related genes; IF related genes; NET-related differentially expressed genes; WGCNA brown module genes; NET-WGCNA-DEGs overlapping genes; Important genes by LASSO; Important genes by SVM-RFE; Important genes by RF; and Key genes by 3 models. Table S2. GO and KEGG enrichment analyses of NET-related differentially expressed genes; and Reactome enrichment analysis of key genes. Table S3. Correlation analysis between key genes and immune cells

## Data Availability

In this study, we used the datasets GSE59071, GSE87473, GSE47980, GSE87466, and GSE92415, as well as NET-related datasets and IF-related datasets. All data are publicly available in the Gene Expression Omnibus (GEO) database (https://www.ncbi.nlm.nih.gov/geo/), the Online Mendelian Inheritance in Man (OMIM) database (https://www.omim.org/), and the GeneCards database (https://www.genecards.org/).

## Abbreviations

UC: Ulcerative colitis
UC-IF: UC-associated intestinal fibrosis
ECM: Extracellular matrix
NE: Neutrophil elastase
MPO: Myeloperoxidase
NETs: Neutrophil extracellular traps
TGF-β1: Transforming growth factor-beta 1
UC-DEGs: NET-related differentially expressed genes
NET-DEGs: NET-related differentially expressed genes
DSS: Dextran Sulfate Sodium
HE: Hematoxylin and eosin staining
IHC: Immunohistochemistry
IF: Immunofluorescence
ELISA: Enzyme-Linked Immunosorbent Assay
